# Recent Progress of Fluxgate Magnetic Sensors: Basic Research and Application

**DOI:** 10.3390/s21041500

**Published:** 2021-02-22

**Authors:** Songrui Wei, Xiaoqi Liao, Han Zhang, Jianhua Pang, Yan Zhou

**Affiliations:** 1 Institute of Microscale Optoelectronics, Shenzhen University, Shenzhen 518060, China; weisongrui@szu.edu.cn (S.W.); hzhang@szu.edu.cn (H.Z.); 2 College of Physics and Optoelectronic Engineering, Shenzhen University, Shenzhen 518060, China; xiaoqiliao@szu.edu.cn; 3 Ångström Laboratory, Department of Engineering Sciences, Uppsala University, 75121 Uppsala, Sweden; 4 Shenzhen Institute of Guangdong Ocean University, International Biological Valley, Dapeng District, Shenzhen 518060, China; njpjh@sina.com; 5 School of Science and Engineering, The Chinese University of Hong Kong, Shenzhen 518172, China

**Keywords:** magnetic sensor, fluxgate, noise, sensitivity, calibration

## Abstract

Fluxgate magnetic sensors are especially important in detecting weak magnetic fields. The mechanism of a fluxgate magnetic sensor is based on Faraday’s law of electromagnetic induction. The structure of a fluxgate magnetic sensor mainly consists of excitation windings, core and sensing windings, similar to the structure of a transformer. To date, they have been applied to many fields such as geophysics and astro-observations, wearable electronic devices and non-destructive testing. In this review, we report the recent progress in both the basic research and applications of fluxgate magnetic sensors, especially in the past two years. Regarding the basic research, we focus on the progress in lowering the noise, better calibration methods and increasing the sensitivity. Concerning applications, we introduce recent work about fluxgate magnetometers on spacecraft, unmanned aerial vehicles, wearable electronic devices and defect detection in coiled tubing. Based on the above work, we hope that we can have a clearer prospect about the future research direction of fluxgate magnetic sensor.

## 1. Introduction

Sensors are devices for detecting, collecting and transmitting various kinds of information from the environment. It is the first step of most forms of artificial intelligence and they play more and more important roles in many fields of industry and daily life. Among them, magnetic sensors detect magnetic fields and currents and are widely used in astro-observation, geophysics observation, non-destructive testing and wearable intelligent devices, etc. [1,2,3,4,5]. To date, many types of magnetic sensors based on different mechanisms have been developed. The magnetic sensors based on fluxgate, Hall effect and magnetoresistance effect are the most widely investigated [5]. In this review, we will focus on the recent progress of fluxgate magnetic sensors regarding both basic research and applications.

**Figure 1:**
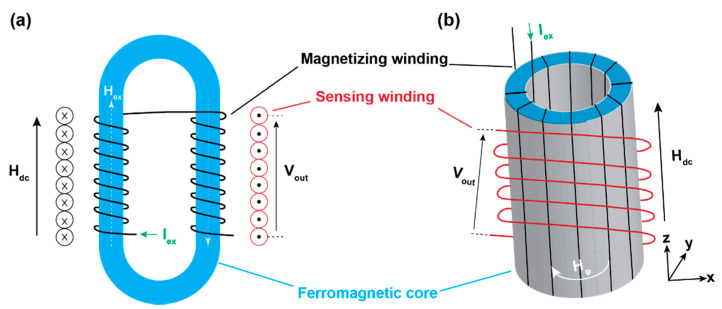
The schematical diagram of: (**a**) parallel fluxgate magnetic sensor and (**b**) orthogonal fluxgate magnetic sensor. The word “parallel” and “orthogonal” in the name mean the orientation between the excitation magnetic field and the probed magnetic field. In (**a**), the excitation magnetic field H_ex_ is parallel to the probed magnetic field H_dc_. In (**b**), the excitation current is along the axial direction. So, the excitation magnetic field is around the circle and orthogonal to the probed field H_dc_. Only the orthogonal fluxgate sensor needs the DC exciting current which should be sufficiently larger than the AC exciting current.

Compared with other magnetic sensors, fluxgate magnetic sensors have advantages such as high sensitivity, high accuracy, high resolution, simple and compact structures, and low noise [6,7,8,9,10,11,12,13,14]. What’s more, they can be used in different kinds of environment and even hostile environments, and they have important applications in weak magnetic field measurements. The basic structures of parallel and orthogonal fluxgate magnetometers are schematically shown in Figure 1 [15]. They are mainly composed of three parts: the magnetizing windings, the sensing windings and the core. The basic working principle of a fluxgate sensor is about the periodic change of the magnetic permeability of the soft ferromagnetic core which is driven by a periodic exciting current. The periodic current in the exciting windings will induce a periodic magnetic field in the magnetic core and this will further induce a periodic current in the sensing windings. When there is no ambient magnetic field, the current of the exciting windings and the sensing windings matches. However, when the soft core is exposed to an ambient magnetic field, the soft core will be more easily saturated in the ambient magnetic field’s direction and less easily saturated in the opposite direction. In this case, the currents of the exciting windings and the sensing windings do not match. The difference between the current of exciting windings and sensing windings are used to estimate the strength of the magnetic field and the output signal is usually integrated to the form of a voltage. For the orthogonal fluxgate sensor, the case is a little different. The noise of orthogonal fluxgate sensor is mainly originated from the domain walls movement. This is the so called Barkhausen noise. To reduce the noise, a DC excitation current which is sufficiently larger than the AC excitation current is applied to reduce the movement of the domain walls. This describes fundamental mode orthogonal fluxgates (FM-OFG).

**Figure 2:**
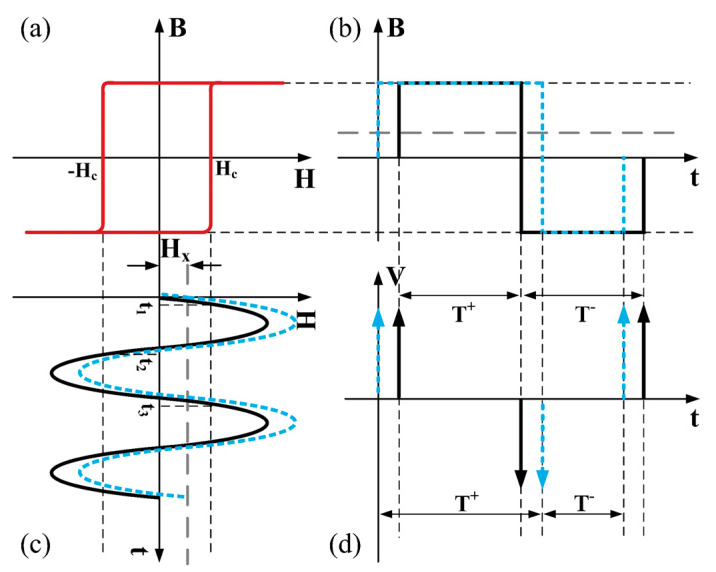
Schematic diagram of the working principle of RTD fluxgate magnetometer. (**a**) Ideal square hysteresis loop, (**b**) magnetic induction intensity corresponding to the hysteresis loop in (**a**), (**c**) sinusoidal excitation signal of an RTD fluxgate with and without a target magnetic field, and (**d**) induced voltage signal from sensing coil with and without a target magnetic field.

To date, many kinds of fluxgate sensors have been developed. According to the orientation between the magnetic field of excitation current and target magnetic field, fluxgate sensors can be divided into parallel fluxgate sensors and orthogonal fluxgate sensors, as shown in Figure 1. They are named after the orientation between the excitation magnetic field and the probed magnetic field. According to the measured signal, they can be divided into voltage fluxgate sensors and time domain fluxgate sensors. In voltage fluxgate sensors, the voltage on the sensing windings is measured to estimate the ambient field. In time domain fluxgate sensors, the time difference between the positive and negative signal will be used to estimate the ambient field. The working principle of the voltage fluxgate sensor is described in the last paragraph. The working principle of the residence time-difference (RTD) fluxgate magnetometer is schematically shown in Figure 2 [16]. Figure 2a is an ideal square hysteresis loop. When there is noexternal magnetic field, the relationship between the magnetic field and time will be shown as the solid black line in Figure 2b. The time intervals between the positive and negative saturation states are equal. Namely, RT D = T^+^ − T^−^ = 0. When an external magnetic field in applied on the magnetic core, the figure will be lifted in the H direction and the equilibrium is broken as shown in Figure 2c. Under this condition, the time difference is no longer zero as shown in Figure 2d.

Their high cost of fabrication has limited the application of fluxgate sensors in more daily necessity fields. The recent trend of fluxgate sensors in industry is to reduce the fabrication cost. Many new fabrication methods have been proposed and the pad-printing technique is one of them. The pad-printing technique is one of the micro-printing techniques which have the advantages of being fast and simple. The micro-printing technique provides new features such as flexibility to fluxgate magnetic sensors. Compared with other micro-printing techniques, pad-printing has advantages such as high printing rates (more than 1500 prints per hour), fast drying of the motif, mass production, precise layer-by-layer printing, and the option of printing on non-flat surfaces [17,18,19]. The structure of this review is arranged as follows: Firstly, it is divided into two main parts, covering basic investigations and applications. In the basic investigation part, recent works about the noise, calibration method and the sensitivity are discussed. For the application part, applications in astro-observation, geographical observation, wearable electronic devices and non-destructive testing are discussed.

## 2. Basic Research

### 2.1. Noise

Butta et al. investigated the relationship between the noise of an orthogonal fluxgate and the composition of the wire-core [20]. The noise of a fundamental mode orthogonal fluxgate is usually ascribed to Barkhausen noise [21,22]. The origin of this noise is the movement of the domain wall in the alloy wires which make up the core of magnetic sensor. To date, most works are devoted to eliminating this kind of noise and much progress has been made. Researchers have applied various methods such as optimizing the geometry to reduce the demagnetization and using multiple wires and the Barkhausen noise has thus been reduced to a very low level [23], but the total noise is still in a high level and the origin of the remaining noise is unclear. In [20], Butta et al. proposed that the remaining noise may come from the magnetostriction of the wire. According to the definition of magnetostriction, on one hand, when an external magnetic field is applied on magnetic materials, there will be a deformation on the direction of the magnetic field. On the other hand, when stress is applied on the magnetic material, the magnetization of the material will also be changed. In the production process of alloy wires, many kinds of stress are introduced. What’s more, when the magnetic sensor is working, stress can also be induced because of the thermal expansion of the wire and the substrate. This means that for a certain magnetic field, there will be different magnetizations in the wire due to the stress and magnetostriction. The fluctuation of the magnetization under a constant external field, by definition, is the noise. As the magnetostriction is usually dependent on the composition of the magnetic materials, in this work different compositions of (Co_1−*x*_Fe*_x_*)_75_Si_15_B_10_ systems were investigated in which *x* = 0.05, 0.055, 0.06, 0.062, 0.065, 0.07 and 0.08. It is found that the noise is really dependent on the composition and the noise reaches the minimum when *x* = 0.06. At the same time, the magnetostriction is also almost vanishing. On the other hand, when *x* = 0.06, the noise does not depend on the stress anymore. It means that this part of noise is strongly dependent on the magnetostriction. Based on the above results, they propose that when annealing, the materials should not be bend and the stress should be as small as possible. That is because the magnetostriction is dependent on the temperature. Even materials without obvious magnetostriction effects at room temperature, may still have strong magnetostriction at annealing temperature.

**Figure 3:**
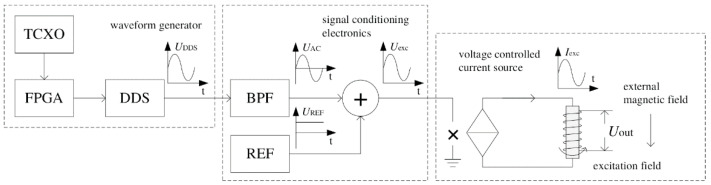
The structure of the excitation module which provides the excitation current in S.X. Song et al.’s work.

Song et al. tried to reduce the noise of the fluxgate sensor system by another way. As the noise is dependent on the input excitation current, they proposed a method to easily find the most proper excitation current for a specific fluxgate sensor [24]. The considered parameters of the excitation current include IAC, IDC and the frequency. The excitation module which provides the excitation current mainly includes three parts: the waveform generator, the signal conditioning electronics and the voltage controlled current source, as shown in Figure 3. With such a module, the proper parameters of the excitation current with the lowest noise can be easily obtained. Finally, they tested the noise of the corresponding sensor in practical application.

Janosek et al. developed the lowest noise fundamental-mode, orthogonal fluxgate magnetometer published so far [25]. Low noise magnetometers are important in many fields such as geomagnetic observatories, scientific experiments in aerospace, nondestructive testing and evaluation, and nanoparticle detection [26,27,28,29,30,31,32,33,34]. According to the structure, fluxgate magnetometers can be classified into the parallel type and orthogonal type. For the parallel type fluxgate magnetometer, the 1-pT noise for 1 Hz can be only obtained with special arrangements and cross correlation measurements [34,35,36,37,38,39]. On the other hand, for the orthogonal type, low noise can be only obtained with the fundamental-mode operated fluxgate. Since the discovery of the orthogonal type by Sasada in 2001 [40], the noise of this kind of magnetometer has been continuously improved. However, a widely acknowledged disadvantage of orthogonal type fluxgate magnetometers is their relatively large offset drift. Although this offset drift can be reduced in either digital or analog domain, in Janosek’s work, this method is not applied because it will cause an increase of the noise.

**Figure 4:**
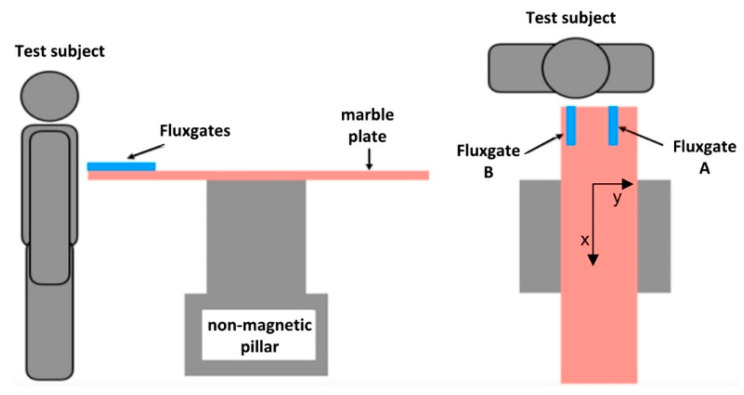
The schematic diagram of MCG measurements based on fluxgate magnetometers.

In Janosek’s work, the noise for 1 Hz is 0.75 and 1.5 pT_rms_/√Hz for the open loop and closed loop, respectively. Additionally, benefitting from the annealing process of the alloy core, the offset drift is also very small (about 2.5 nT/K). With such a good property, the developed fluxgate magnetometer has many promising applications. For example, compared to a low-noise observatory magnetometer, it is found that it has good performance at mHz frequency and is suitable for the measurement such as magnetotellurics. It can be also used in magnetocardiography (MCG) measurement. Benefitting from the low noise and offset drift, MCG measurements can be performed under room temperature and without shielding and averaging. The arrangement of the MCG measurement is shown in Figure 4 [25].

### 2.2. Errors and Calibration Methods

**Figure 5:**
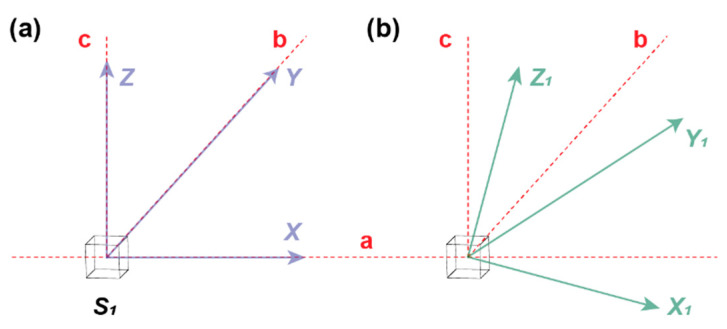
The difference between ideal coordinate (**a**) and real coordinate (**b**) about the orthogonal error. The a-b-c coordinate system is the reference coordinate. Under ideal condition, the output coordinate system coincides with the reference coordinate, as shown in Figure 4a. However, under real condition, there will be a deviation as shown in Figure 4b. For Equation (3), *α_x_*, *β_y_* and *γ_z_* are the angle between *x* and *x*_1_, *y* and *y*_1_, *z* and *z*_1_, respectively. Accordingly, *α_y_*, *α_z_*, *β_x_*, *β_z_*, *γ_x_*, and *γ_y_* are the angle between *x* and *y*_1_, *x* and *z*_1_, *y* and *x*_1_, *y* and *z*_1_, *z* and *x*_1_, *z* and *y*_1_, respectively.

For tri-axis orthogonal fluxgate magnetometers, there are three kinds of inevitable errors because of the limitation of manufacture technology: zero position error, sensitivity error and orthogonal error. For the zero position error, it means the output of the magnetometer is not zero under a zero magnetic field. If we only consider the effect of core remanence and circuit drift, the relationship between the output of a real magnetometer, ideal magnetometer and zero position error term can be written as:

(1) $$\left[\begin{array}{c} B_{x1}\\ B_{y1}\\ B_{z1} \end{array}\right]=\left[\begin{array}{c} B_{x}\\ B_{y}\\ B_{z} \end{array}\right]+\left[\begin{array}{c} B_{x0}\\ B_{y0}\\ B_{z0} \end{array}\right],$$

in the above equation, *B_x_*_1_, *B_y_*_1_ and *B_z_*_1_ are real output. *B_x_*, *B_y_*, and *B_z_* are ideal output. *B_x_*_0_, *B_y_*_0_ and *B_z_*_0_ are the zero position error terms and they can be obtained by the output of the magnetometer when there is no magnetic field. The sensitivity error is originated from the difference between the sensitivity of the three coordinate axes. The relationship between the real output, ideal output and the sensitivity of three axes can be written as:

(2) $$\left[\begin{array}{c} B_{x1}\\ B_{y1}\\ B_{z1} \end{array}\right]=\left[\begin{array}{ccc} K_{x} & 0 & 0\\ 0 & K_{y} & 0\\ 0 & 0 & K_{z} \end{array}\right]\left[\begin{array}{c} B_{x}\\ B_{y}\\ B_{z} \end{array}\right],$$

in the above equation, *B_x_*_1_, *B_y_*_1_ and *B_z_*_1_ are the real output. *B_x_*, *B_y_*, and *B_z_* are the ideal output. *K_x_*, *K_y_*, and *K_z_* are the sensitivities of the *x* axis, *y* axis and *z* axis, respectively. In ideal case, *K_x_* = *K_y_* = *K_z_* = 1. The orthogonal error is originated from the mismatch between the ideal coordinate and real coordinate or the relationship between the real coordinates are not exactly orthogonal. As above, if the angle between the ideal coordinate and real coordinate is as shown in Figure 5 [41], the relationship between the real output and ideal output can be written as:

(3) $$\left[\begin{array}{c} B_{x1}\\ B_{y1}\\ B_{z1} \end{array}\right]=\left[\begin{array}{ccc} \cos\alpha_{x} & \cos\alpha_{y} & \cos\alpha_{z}\\ \cos\beta_{x} & \cos\beta_{y} & \cos\beta_{z}\\ \cos\gamma_{x} & \cos\gamma_{y} & \cos\gamma_{z} \end{array}\right]\left[\begin{array}{c} B_{x}\\ B_{y}\\ B_{z} \end{array}\right],$$

in ideal case, *α_x_* = *β_y_* = *γ_z_* = 0, *α_y_* = *α_z_* = *β_x_* = *β_z_* = *γ_x_* = *γ_y_* = 90°. If we consider a fluxgate gradiometer which is composed of two identical fluxgate magnetometers, an additional error term will occur which is originated from the inconsistent placement of two fluxgate magnetometers. It is named the position error for the fluxgate gradiometer and the form of the expression is same to the orthogonal error.

In Xu et al.’s work, the effect of sensitivity error, orthogonal error and position error are considered together in one matrix ***A*** and the zero position error is considered in another term ***B*_0_**. So, the total effect of these error terms can be written as:

(4) $$B_{1}=A\cdot B+B_{0},$$

where $B_{1}=\left[\begin{array}{c} B_{x1}\\ B_{y1}\\ B_{z1} \end{array}\right]$, $A=\left[\begin{array}{ccc} a_{11} & a_{12} & a_{13}\\ a_{21} & a_{22} & a_{23}\\ a_{31} & a_{32} & a_{33} \end{array}\right]$, $B=\left[\begin{array}{c} B_{x}\\ B_{y}\\ B_{z} \end{array}\right]$, $B_{0}=\left[\begin{array}{c} B_{x0}\\ B_{y0}\\ B_{z0} \end{array}\right]$.

With this method, the calibration is quick and accurate. The coefficients ***A*** and ***B*_0_** can be obtained by the method of undetermined coefficients after several measurements. At the end of the paper, this calibration method is performed in experiments. For a single three-component magnetometer, the maximum deviation can be reduced from 552.4 nT to 15.0 nT with this calibration method. For the fluxgate gradiometer which is composed of two similar magnetometers, the maximum deviation can be reduced from 3891.5 nT to 37.2 nT with this calibration method.

Pan et al. provided a new calibration method for triaxial fluxgate magnetometers (TFMs) which combines a magnetic shielding room (MSR) and a triaxial uniform magnetic field coil (TUMC) [42]. As described above, the TFM has three intrinsic errors. To date, two kinds of calibration methods are proposed. The vector calibration method uses a known vector magnetic field as the reference and its accuracy is strongly dependent on the accuracy of the known magnetic field [43]. However, the disturbance of the environmental magnetic field and the inaccuracy of the turntable cannot support the pursuit of calibration of fluxgate magnetometers, so another method, the scalar calibration method is proposed, whose accuracy is not dependent on the attitude information. The concept of the scalar calibration method was proposed as early as the 1970s. Under ideal conditions, the form of the TFM output should be a spherical surface. However, due to the existence of the above three errors, the form of the TFM output will change from a sphere to an ellipsoid. Based on the shape of the ellipsoid, the magnetometer can be calibrated [44].

**Figure 6:**
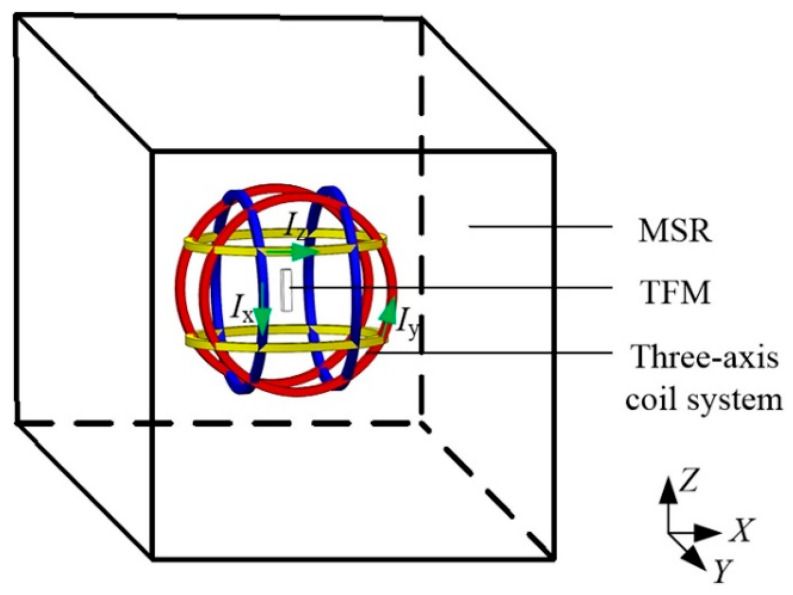
Schematic diagram of the calibration method of TFMs based on MSR and TUMC.

**Figure 7:**
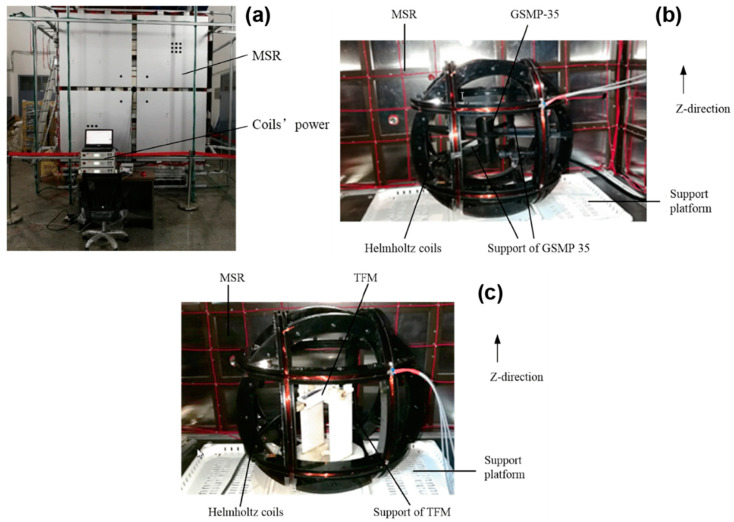
Experimental picture of the device for calibration of TFMs with MSR and TUMC: (**a**) Test platform; (**b**) Calibration of TUMC; (**c**) Calibration of TFM.

In practical calibration, the accuracy may be affected by the environmental magnetic field [45,46] and the intrinsic magnetic impurities in the turntable [47,48] if the turntable is used to tune the attitude of the magnetic field. The problem of the environmental magnetic field is more and more serious with the widely used electronic devices. To solve this problem, in this work, the MSR is used. On the other hand, in some early works, to reduce the magnetic field which is originated from the power system of the turntable, they use a manual method to rotate the turntable, but in this way, another problem about the time evolution of the magnetic field emerges because it usually takes more time to tune the turntable manually. To solve this problem, a standard TUMC is used. In Pan’s work, firstly, they constructed the error model of the TUMC and TFM. Secondly, the magnetic environmental noise model of MSR is added. The most important part of this work is the analysis of the magnetic field disturbance in MSR and the magnetic field characteristics of TUMC in MSR. Finally, experiments are performed and it is proved that this calibration method has high sensitivity and accuracy. The schematic diagram and the experimental picture of this method is shown in Figure 6 and Figure 7, respectively [42].

### 2.3. Sensitivity and Other Related Research Works

Sensitivity is a core property of sensors. For fluxgate magnetometers, it is usually expressed as the change of an output such as voltage or time difference per ambient field. Szewczyk et al. tried to increase the sensitivity of fluxgate sensors produced by the printed circuit board method [15]. The printed circuit board method is proposed to solve the problem of the high cost of traditional fluxgate sensors. The high cost of traditional fluxgate sensors is due to the large amount of manual work involved in their production, so the printed circuit board method can reduce the cost of fluxgate sensors efficiently, but at the same time, the sensitivity of fluxgate magnetometers based on printed circuit boards is strongly reduced [49]. In [15], the authors investigated the feasibility of increasing the sensitivity by lengthening the core and using a magnetic flux concentrator based on the finite element method and open-source software. The magnetic flux concentrator has been applied in many other magnetic sensors to increase the sensitivity [50,51]. Generally speaking, it increases the sensitivity by decreasing the demagnetization field. However, the performance of magnetic flux concentrators based on thin layer fluxgate sensors has never been discussed. In this work, it is found that a longer core will effectively increase the sensitivity but the effect of the magnetic flux concentrator is very weak, so it is not recommended to use a magnetic flux concentrator in a printed circuit board-based fluxgate magnetometer.

**Figure 8:**
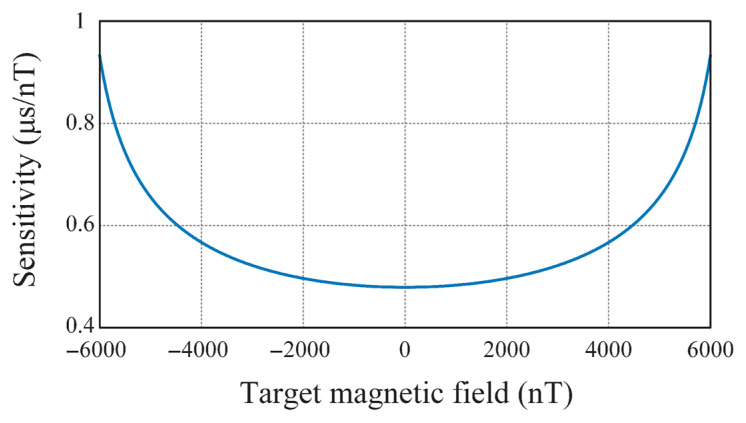
Relationship between sensitivity of RTD fluxgate magnetometer and target magnetic field. Because of this relationship, the RTD fluxgate magnetometer can only be applied in detecting weak magnetic field.

To increase the sensitivity of a RTD fluxgate magnetometer, Chen et al. built a sensitivity model for RTD fluxgate magnetometers [16]. The model is clear and simple and can discuss the sensitivity of the RTD fluxgate and the coercivity of the magnetic core separately. When the excitation current is sinusoidal, the sensitivity of the RTD fluxgate sensor can be written as the function of coercivity of the magnetic core as well as amplitude and frequency of the driving field, as shown in Equation (5) [52]:

(5) S=∂RTD∂Hx=2ω[1Hem1−(Hc+HxHem)2+1Hem1−(Hc−HxHem)2],

However, as the coercivity of the core is dependent on the driving condition and the relationship between them is unknown, it is difficult to calculate the sensitivity with Equation (5). What’s more, the sensitivity is also dependent on the target magnetic field as shown in Figure 8 [16]. It can be seen that only under low target magnetic field conditions, the sensitivity is nearly uniform (within ± 1000 nT), so the sensor can only accurately measure low fields [53]. In Szewczyk’s work, they deduced the expression of sensitivity under sinusoidal excitation from the expression of sensitivity under trilinear excitation and tried to calculate the differential permeability µd more accurately. Finally, the model is verified by experiments. This work separates the investigation of sensitivity of RTD fluxgates and coercivity of the magnetic core. It also offers a platform to further investigate the structure of RTD fluxgate magnetic sensors.

Yang et al. proposed a time domain method to measure current [54]. It is a bidirectionally saturated fluxgate based on open-loop self-oscillating technology. The advantage of this method is that there is no time delay because the current is estimated by the time difference between the first half-cycle and the second half-cycle of the excitation. To obtain a better temperature stability, the authors used a nanocrystalline alloy core. Finally, the performance of the method is confirmed by experiments and the properties such as linearity and sensitivity are discussed. Ramasamy et al. characterized the superconducting quantum interfere device (SQUID)-based time domain electromagnetic (TDEM) system [55]. It is found that the early time of the fluxgate magnetometer behaves like a combination of induction coil and magnetic field sensor. However, at later times, it behaves like a pure magnetic field sensor.

## 3. Applications

Because of their high sensitivity and resolution, fluxgate magnetic sensors are widely used in geophysics and astro-observations. Recently, China’s first Mars mission Tianwen-1 and NASA’s spacecraft Juno both used fluxgate sensors to detect the magnetic field on Mars and Jupiter. The unmanned aerial vehicles are also used as the new carrier to perform such detections about Earth’s magnetic field apart from the traditional carriers such as a helicopter aeromagnetic surveys or a ground magnetic surveys.

### 3.1. Astro Observation

**Figure 9:**
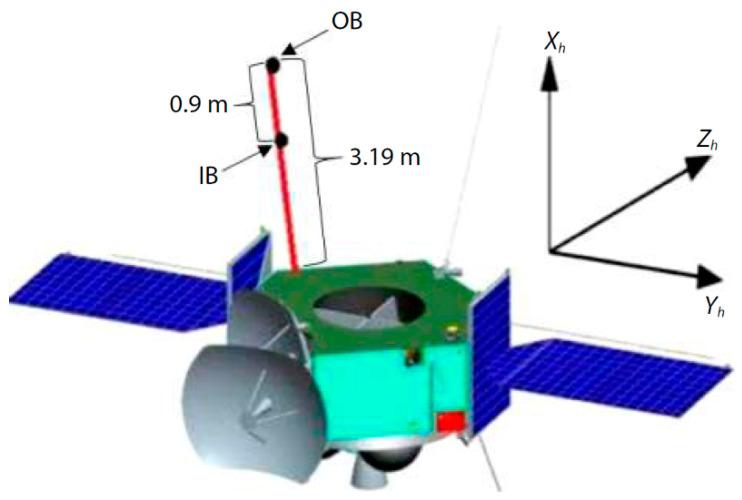
The schematic diagram of Tianwen-1 Mars orbiter and the dual-structure Mars Orbiter Magnetometer on it.

Recently, China’s first Mars mission Tianwen-1 was lunched and Mars Orbiter Magnetometer (MOMAG) is one of the seven scientific payloads on it. The project is performed by a team from Chinese Academy of Sciences Key Laboratory of Geospace Environment of University of Science and Technology of China [56]. The MOMAG includes two separate fluxgate sensors which are fixed on a boom as shown in Figure 9 [56]. The dual magnetometer structure is designed to eliminate the interference of the magnetic field induced by the orbiter. Considering that the distance between the two magnetometers (about 0.9 m) is much smaller compared with the size of Mars’s magnetosphere, so the difference of the results between the two magnetometers can be assumed to be solely due to the orbiter and the corresponding noise. In this way, the magnetic field from the orbiter can be separated from the total magnetic field. This method is firstly developed in the Venus Express project in 2006.

**Figure 10:**
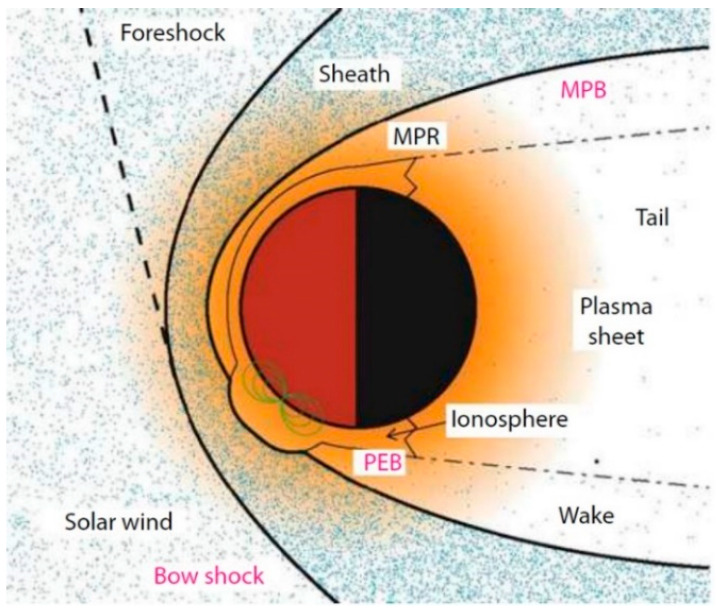
Schematic diagram of the magnetosphere produced by solar wind on Mars.

It has been found that there is no global magnetic field on Mars by the Mars Global Surveyor (MGS) Mission in 1998, but two other kinds of magnetic field still exist. They are the magnetosphere produced by the solar wind and the local strong magnetic crustal field near the southern highlands of Mars. The most important mission of MOMAG is to measure the vector magnetic field of the two sources and the interaction between them. The solar wind is a cluster of plasmas running under supersonic speed. It carries the interplanetary magnetic field (IMF) with it. As it cannot pass through Mars, it leaves a magnetosphere around the Mars with the frozen-in IMF. The structure of the magnetosphere is shown in Figure 10 [56]. It mainly includes the bow shock, magnetosheath, magnetic pileup boundary (MPB) and the ionosphere or photoelectron boundary (PEB). The bow shock is the outermost part of magnetosphere. The solar wind transitions from supersonic to subsonic when it passes through the bow shock. The layer inside the bow shock is the hotter, denser, more turbulent magnetosheath and the MPB is the lower boundary of magnetosheath. The layer inside the magnetosheath is the PEB. It is the critical part of magnetosphere of Mars which separates the ions mostly from the solar wind and the ions mostly from Mars. On the night side, there is a magnetic tail. About the magnetic crustal field near the southern highlands, we know that it is much stronger than the magnetic field of the Earth. It forms a small local magnetosphere and strongly affects the magnetic field from solar winds at lower altitude. On one hand, it can protect the planet surface from the damage of electrically charged particles in universe. On the other hand, it can also shield the ion escape flux near the southern highlands.

As the fluxgate magnetometers do not perform absolute measurement, they need to be calibrated both on Earth and in-flight. After the measurement, the results need to be further corrected to exclude the various influencing factors. To save the limited power of the spacecraft, the observation plan is also carefully designed. Near the periareon and apoareon, the magnetometer will work at the sampling frequency of 32 Hz. In other positions, the magnetometer will work at the sampling frequency of 1 Hz. On average, the magnetometer will detect the magnetic field of Mars’s magnetosphere 1.89 kilobits per second.

**Figure 11:**
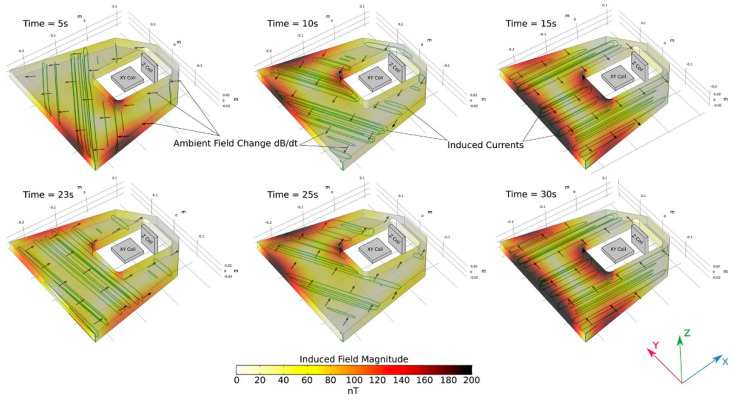
The results of different times of the finite element method used in analyzing the magnetic field caused by the spinning space craft.

**Figure 12:**
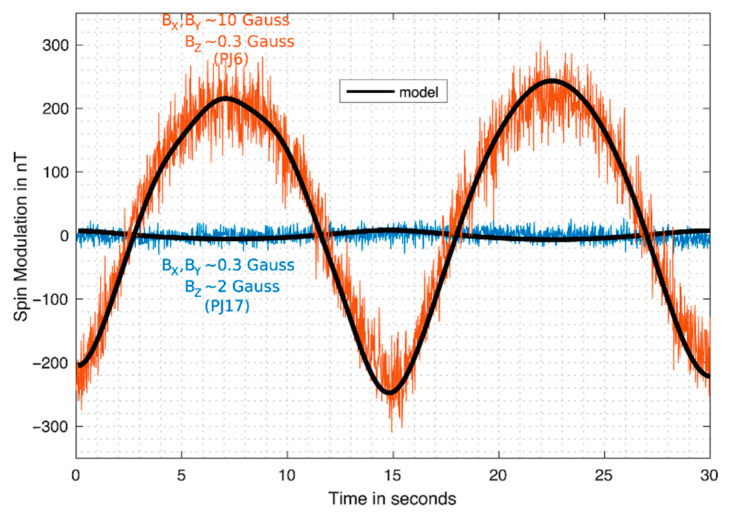
Two different cases of the modulation results. The blue one is for the case with a stronger magnetic field in *z* direction while the yellow one is for the case with a stronger magnetic field in *x*-*y* direction.

Kotsiaros et al. reported the measurement of Jupiter’s magnetic field by the fluxgate magnetometer installed on the spinning spacecraft Juno [57]. Similar to Tianwen-1’s design, the fluxgate magnetometer on Juno also uses two independent magnetometers placed on a bar of the spacecraft to eliminate the effect of magnetic field induced by the spacecraft itself. Differently, Juno spins every 30 s, or about two turns per minute. According to Faraday’s law of electromagnetic induction or Lenz’s law, a spinning conductor in magnetic field will induce an edge current in the conductor and the edge current will in turn generate an additional magnetic field. This additional magnetic field can be seen as the background noise and it will affect the measurement of the Jupiter’s intrinsic magnetic field. According to author’s estimations, the additional magnetic field is about 0.001 Gauss in an intrinsic field of about 3 Gauss. For fluxgate sensors, this value is pretty large and should not be ignored. The authors applied the finite element method and the Maxwell equations to quantitatively calculate the spinning induced magnetic field. They also put up a method to eliminate this additional field. The corresponding results are shown in Figure 11 and Figure 12 [57]. At the end of the paper, they also provide another method which is similar to the “thin shell” method to evaluate the induced field.

### 3.2. Geophysics Observation

Le Maire et al. applied a fluxgate sensor which is mounted on an unmanned aerial vehicle (UAV) in magnetic mapping [58]. Compared with other geophysical mapping methods, magnetic mapping has the advantages of mapping both large scale objects and small-scale objects with the same instrument. Traditionally, magnetic mapping is usually performed by the ground or airborne magnetic surveying. However, there is a gap of height between the ground and airborne magnetic surveying because the fixed-wing aircraft and helicopters cannot fly too low for safety reasons. For ground magnetic surveying, its result can be easily affected by the noise from the anthropogenic origin which will affect the results of airborne magnetic surveying, so the results of ground and airborne magnetic surveying are not easy to relate. However, the demand for continuously measuring the magnetic field of different heights is strong. It is well known that the map obtained by magnetic method can vary by several orders of magnitude according to different distances to the magnetic origin or different spacing between magnetic profiles.

The recently emerging UAV is an ideal solution for this problem. As UAVs can fly at the height between ground and 100 m, it effectively fills the gap of traditional ground and airborne magnetic surveying. Generally, the UAV can be divided into two types: fixed wing and rotary wing. The fixed wing UAVs have higher speed and larger range. But they need larger plate to take off and land. On the other hand, the rotary wing UAVs is a new member of the family of UAVs used for airborne magnetism. It is smaller and more maneuverable comparing with the fixed wing UAVs. It only requires a very small plate to take off and land. But at the same time, the spatial range of rotary wing UAV is also smaller (several kilometers) and the speed is lower (typically 10 m/s). In this work, as the rotary wing UAV is used, the smaller and lighter fluxgate vector magnetometer is more preferrable. As the fluxgate vector magnetometer does not measure the absolute magnetic field, it needs to be calibrated before using. In this work, the noise is mainly from the intrinsic and induced magnetic field of UAV.

**Figure 13:**
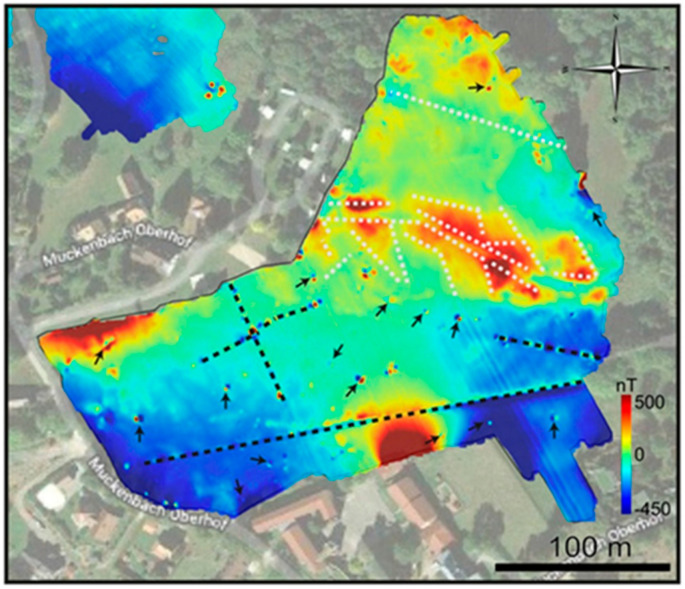
The magnetic map obtained by the fluxgate magnetometer on a UAV at the height of 100 m with a satellite image as the background.

**Figure 14:**
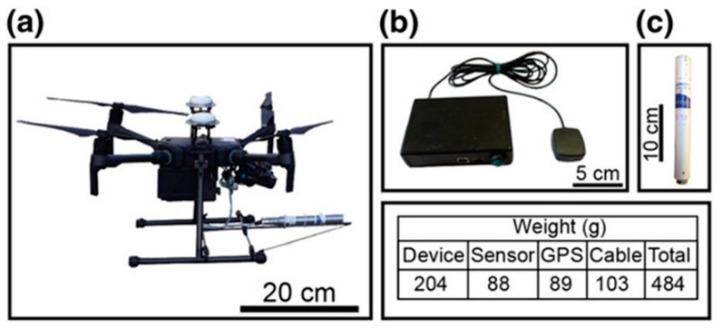
(**a**) The Matrice 210 RTK quadcopter designed by Da Jiang Innovation (**b**) and the Bartington MAG03-MC fluxgate magnetometer (**c**). The overall weight of the magnetic equipment is 484 g.

The work is performed in the Northern Vosges Mountains. The geological context is complex and many anthropogenic objects, such as houses, pipelines and various buried ferrous objects, may affect the local magnetic field. This place has been widely investigated because it is the deep drilling project for geothermal resource prospecting. In this work, the magnetic anomaly map of ground (0.8 m), 1.2 m, 30 m and 100 m are drawn. The results of 100 m after reduction to the pole of ground survey in the south is shown in Figure 13 [58]. It is superimposed on a satellite image. A Matrice 210 RTK quadcopter designed by Da Jiang Innovation (DJI, Shenzhen, China) is used as shown in Figure 14 [58]. The results of ground survey and UAVs are compared and the comparison with upward continuation is also performed. It is found that both the wavelength and amplitude of the magnetic anomaly maps are different for different altitudes. The results are consistent with former studies both in the crustal scale (e.g., identification of major faults or geological contacts) and local scale (e.g., identification of anthropic pipes or archaeological remains).

### 3.3. Other Related Applications

**Figure 15:**
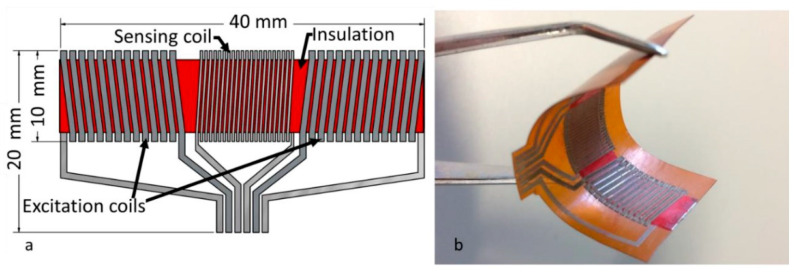
Structure of the printed fluxgate magnetometer on wearable intelligent device (**a**) and the experimental picture (**b**).

**Figure 16:**
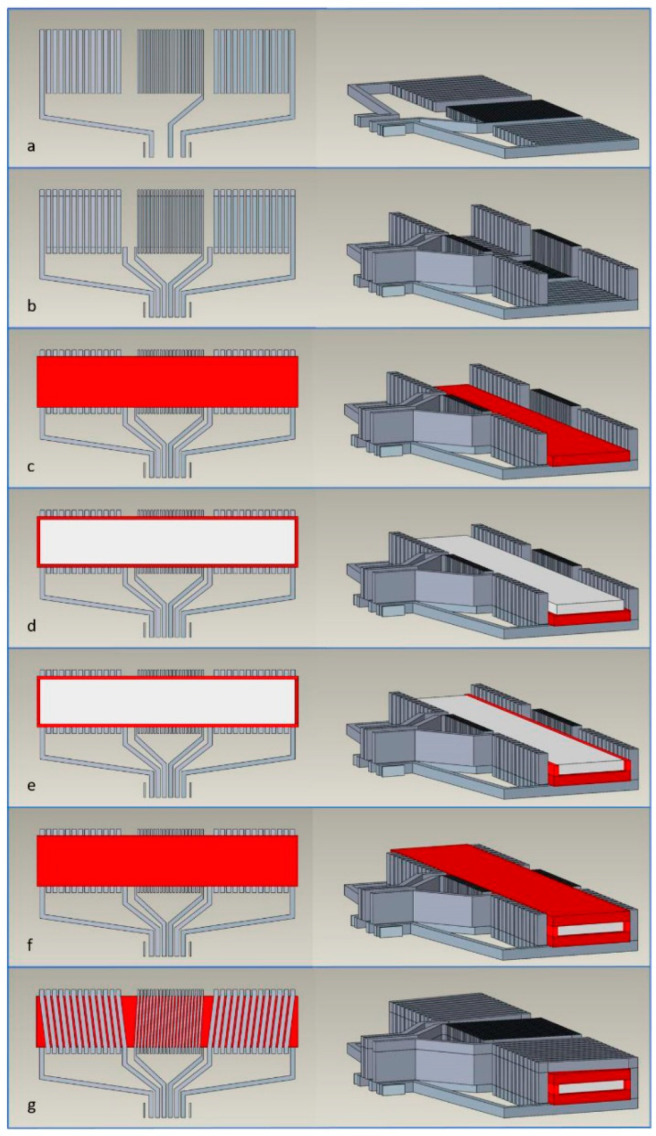
The producing process of the printed fluxgate magnetometer. (**a**) Printing the bottom conductive layer; (**b**) Printing the conductive vias; (**c**) Printing the bottom insulating layer; (**d**) The mounting and attachment of magnetic core; (**e**) Printing the insulating walls; (**f**) Printing the top insulating layer; (**g**) Printing the top conductive layer.

**Figure 17:**
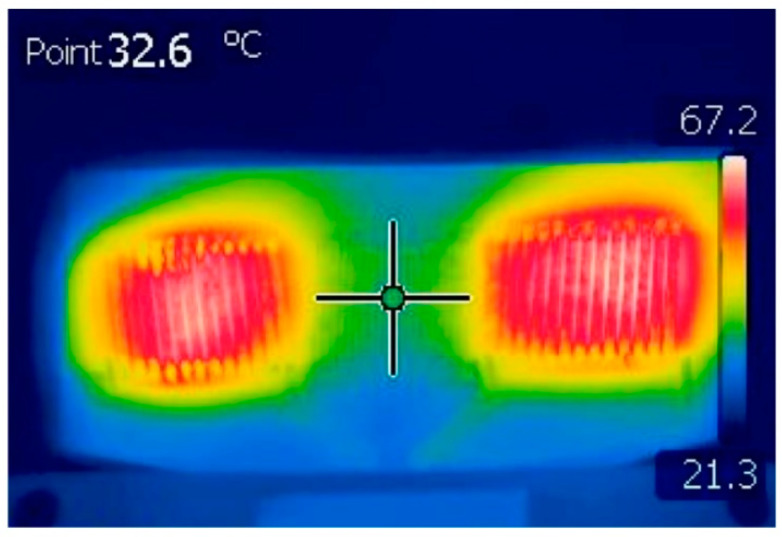
The temperature distribution of the printed fluxgate magnetometer under a certain working condition.

Schoinas et al. fabricated and characterized a flexible fluxgate sensor with pad-printed solenoid coils [59]. Recently, wearable intelligent devices are a new trend in consumer daily electronic devices. In this trend, the flexible sensor is the indispensable component. The fluxgate magnetic sensor is well known for its high sensitivity and resolution for the low magnetic field. However, in consumer electronic devices, the high sensitivity and resolution are not necessary, so it is replaced by other kinds of magnetic sensors such as anisotropic magnetoresistance (AMR) sensors. The main obstacle for wider application of fluxgate sensor is their complex and expensive fabrication method, so a method that can easily fabricate fluxgate sensors in large numbers is urgently needed, even though in this process the sensitivity and resolution may be lowered. In this work, a pad-printing technique is applied to fabricate the solenoid coil of the sensor [60,61,62,63]. The schematic diagram and the photographic view of the printed sensor are shown in Figure 15 [59]. The synthesis process is schematically shown in Figure 16 [59]. After synthesis, the performance of the sensor is characterized. The corresponding results include the waveform of the output voltage of the sensing coil, the magnitude of the sensor’s second harmonic response and the sensor sensitivity. All the above results are measured under different excitation currents. It is worth noting that the noise of the sensor is at a high level. This is due to the shape of the bar sensor which is similar to the case of linear transformer. Additionally, the temperature of the sensor is also measured. It is found that the temperature is dependent on the strength and frequency of the excitation current. The highest temperature is 67.2 °C which appears near the excitation coils under a 700-mA p-p excitation current at 100 kHz and the temperature of the sensing coil is 32.6 °C under the same condition. It can be concluded that the printing technique is imperfect because the temperature distribution is unsymmetric under the same current. The corresponding results are shown in Figure 17 [59].

Apart from the fluxgate magnetometer which needs the AC or DC excitation current, Zhou et al. applied a fluxgate magnetometer based on weak magnetic detection technology to probe the defects of arbitrary orientations in coiled tubing (CT) [64]. Traditionally, magnetic flux leakage (MFL) testing is one of the non-destructive testing (NDT) methods used for detection CT failures [65,66,67]. However, it is only sensitive to circumferentially distributed defects and cannot detect axially distributed defects. The method proposed in this work can effectively detect the defects in any direction. It can also distinguish between different types of defects such as axial, circumferential and pore type defects. Additionally, as it does not require the rotation of magnetic fields, the size of the equipment is relatively small.

**Figure 18:**
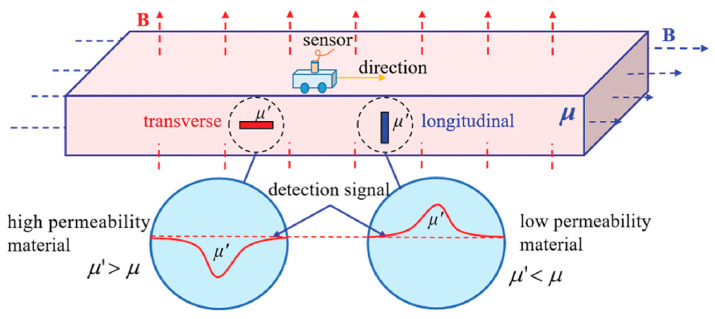
The working principle of the weak magnetic detection technology.

The working mechanism of the weak magnetic field detection technology in defect detection is schematically shown in Figure 18 [64]. The mechanism of the technology is that the permeabilities of the workpiece (*µ*) and the defect (*µ′*) are usually different. If the permeability of the defect is higher (*µ′* > *µ*), the curve of magnetic induction intensity ***B*** will be concave as shown on the left bottom panel of Figure 18. If the permeability of the defect is lower (*µ′* < *µ*), the curve of magnetic induction intensity ***B*** will be upward as shown on the right bottom panel of Figure 18. The magnetic induction intensity of every position around the workpiece can be measured by passing a three-dimensional magnetic sensor above the workpiece. Then, the gradient of magnetic induction intensity with space can be obtained by $\frac{\partial B}{\partial x}\approx \frac{B\left(x+\Delta x\right)-B\left(x\right)}{\Delta x}$. As Δx is a constant, $\frac{\partial B}{\partial x}$ and B(x+Δx)−B(x) should have the same shape but different amplitudes, so the interference between adjacent magnetic anomalies will be reduced and this is helpful to distinguish the superimposed magnetic anomalies. In addition, comparing with the gradient of magnetic induction intensity of the workpiece, the gradient of the geomagnetic field is much smaller, so it can suppress the local background gradient field. If we calculate the gradient of the three components of ***B*** (*B_x_*, *B_y_*, *B_z_*) in three directions (*x*, *y*, *z*), then the magnetic gradient tensor *G* can be obtained as Equation (6):

(6) $$G=\left[\begin{array}{ccc} \frac{\partial B_{x}}{\partial x} & \frac{\partial B_{x}}{\partial y} & \frac{\partial B_{x}}{\partial z}\\ \frac{\partial B_{y}}{\partial x} & \frac{\partial B_{y}}{\partial y} & \frac{\partial B_{y}}{\partial z}\\ \frac{\partial B_{z}}{\partial x} & \frac{\partial B_{z}}{\partial y} & \frac{\partial B_{z}}{\partial z} \end{array}\right]=\left[\begin{array}{ccc} \frac{\partial^{2}\varphi_{m}}{\partial x^{2}} & \frac{\partial^{2}\varphi_{m}}{\partial x\partial y} & \frac{\partial^{2}\varphi_{m}}{\partial x\partial z}\\ \frac{\partial^{2}\varphi_{m}}{\partial y\partial x} & \frac{\partial^{2}\varphi_{m}}{\partial y^{2}} & \frac{\partial^{2}\varphi_{m}}{\partial y\partial z}\\ \frac{\partial^{2}\varphi_{m}}{\partial z\partial x} & \frac{\partial^{2}\varphi_{m}}{\partial z\partial y} & \frac{\partial^{2}\varphi_{m}}{\partial z^{2}} \end{array}\right]=\left[\begin{array}{ccc} B_{xx} & B_{xy} & B_{xz}\\ B_{yx} & B_{yy} & B_{yz}\\ B_{zx} & B_{zy} & B_{zz} \end{array}\right],$$

where *φ_m_* is the magnetic scalar potential and its differential with space is the corresponding magnetic induction intensity in that direction. *B_ij_* (*i*, *j* = *x*, *y*, *z*) are the components of the tensor in the *j* direction along the *i* axis. The magnetic gradient tensor contains the information of the defects. In Zhou’s work, three magnetometers which are responsible for one direction (*x*, *y* and *z*), respectively, are composed orthogonally together and form the triaxial fluxgate sensor. Similar to Section 2.2 of this review, they also calibrate the zero error, scale error and orthogonal error of the triaxial system. At last, they applied this method in experiment and found that different forms and directions of defects can be distinguished effectively.

## 4. Summary and Outlook

In the past two years, much progress has been made about lowering the noise, new calibration methods and increasing the sensitivity of fluxgate magnetometers. Fluxgate magnetic sensors have been applied in many new fields and many practical problems solved. In the future, we believe that the following research fields will be productive. Firstly, the work about lowering the noise should focus on the type of noise apart from the Barkhausen noise because after years of effort, the Barkhausen noise has been reduced to a low level. More attention should be paid to the quality of the alloy core because the remanent noise may be due to the magnetostrictive effect of the alloy core. Additionally, the offset drift is also dependent on it. Secondly, due to the lower noise and wider bandwidth, the fundamental mode is a more popular trend comparing with the second harmonic mode. Thirdly, time domain magnetometers such as residence time difference fluxgate magnetometers are a new trend of this field. Fourthly, the most important problem of fluxgate magnetometers in consumption applications is their high cost, so the main target of this field is to reduce the production cost, even though in this process, the sensitivity may be reduced and the noise may be increased to some extent.

## Data Availability

No new data were created as it is a review paper.
